# A naturalistic analysis of rTMS treatment outcomes for major depressive disorder in West Australian youth

**DOI:** 10.3389/fpsyt.2025.1513339

**Published:** 2025-03-04

**Authors:** Aleksandra Miljevic, Kyle Hoath, Kerry S. Leggett, Lauren A. Hennessy, Caitlan A. Boax, Jaroslaw Hryniewicki, Jennifer Rodger

**Affiliations:** ^1^ Brain Plasticity, Perron Institute for Neurological and Translational Science, Nedlands, WA, Australia; ^2^ Modalis TMS, Stirling, WA, Australia; ^3^ School of Biological Sciences, University of Western Australia, Crawley, WA, Australia

**Keywords:** TMS, rTMS, adolescent, youth, depression, anxiety, stress

## Abstract

**Objective:**

Repetitive transcranial magnetic stimulation (rTMS) is an effective, evidence-based treatment for major depressive disorder (MDD) in adults and is publicly funded in Australia. However, there is limited data as to its efficacy and safety in treating MDD in adolescent and youth populations.

**Methods:**

This retrospective report examined routinely collected data of 46 outpatients aged 17 to 25 years old, who received rTMS treatment for MDD in a private TMS clinic. Primary outcomes measures were the Montgomery-Asberg Depression Rating Scale (MADRS) and the depression subscale of the 21-item Depression, Anxiety and Stress Scale (DASS-21). Secondary measures included the anxiety and stress sub-scales of the DASS-21, a measure of Quality of Life (QoL) Enjoyment and Satisfaction Questionnaire, and the Cognitive Failures Questionnaire (CFQ).

**Results:**

A 4–7-week course of rTMS significantly reduce symptoms of self-reported depression (42.5% response) and clinician-assessed depression (40.7% response). Both anxiety and stress significantly reduced across the course of rTMS treatment and significant improvements to QoL and self-reported cognition were observed. Reported side effects following rTMS in youth included a mild headache and fatigue.

**Conclusions:**

The findings of this naturalistic report suggest that an acute course of rTMS is safe and effective – resulting in similar response rates in adolescent and youth patients as reported in adults. Future large-scale, randomized, and sham-controlled trials are needed to consolidate and add to these findings.

## 1 Introduction

Depression is one of the most common mental illnesses worldwide and one of the leading contributors of global disease burden (1). It’s estimated that 1 in 7 Australians are affected by depression and that 45% of all Australians have experienced a mental health disorder like depression within their lifetime (2). In addition, Australia has experienced a marked increase in the prevalence of mental health issues among youth [15- to 24-year-olds; (1)]. In 2007, 26% of those aged 16–24 had experienced a mental disorder within the last 12-months; in 2020–2022, this figure increased to 39% (2), likely precipitated by the COVID-19 pandemic and its repercussions (1).

The current pathway to seeking treatment for depression in Australian youth includes psychotherapies usually in combination with pharmacotherapies (3). Whilst moderately effective for most, >30% of adolescents and youth with depression fail to respond to pharmacotherapy treatments (4, 5), and this response is suggested to be even lower for psychotherapies, especially in moderate to severe case of MDD (6, 7). Moreover, pharmacotherapies can produce more frequent and significant side effects in youth compared to adults, including increased suicide ideation (8). Meanwhile, Electroconvulsive Therapy (ECT) is prescribed only in rare cases due to safety concerns of seizure induction in the developing brain (3, 9). Overall, there are few safe, well tolerated, and effective treatment options available for youth with (severe) depression and suicidal ideation. Thus, more favorable, and effective treatment options are needed.

Repetitive Transcranial Magnetic Stimulation (rTMS) is a non-invasive, non-convulsive brain stimulation modality (10). rTMS treatment for depression typically involves the delivery of repetitive, rapid electromagnetic pulses through the scalp to the (left or right) dorsolateral prefrontal cortex (DLPFC) via a handheld device called a ‘coil’ (11). While the exact mechanisms of action of rTMS are not yet fully understood, high frequency stimulation to the left DLPFC and low frequency stimulation to the right DLPFC are proposed to modulate neurotransmission, synaptic plasticity and neural circuits as well as neurogenesis (12, 13), thereby producing an antidepressant effect (14).

rTMS is now recommended as a clinical treatment for depression by the Australian and New Zealand College of Psychiatrists (RANZCP, 2018) and has emerged as a publicly funded, mainstream treatment in clinical practice in adults over the age of 18 years who have failed to respond to 2 or more antidepressant pharmacotherapies. For patients under the age of 18 years, no public funding is available in Australia and a secondary psychiatric opinion is required as well as the consent of both the patient and their legal guardian. In practice, however, rTMS is still predominantly applied to older adults and the results of many prominent randomized controlled clinical trials (RCTs) are based on evidence from patient populations with an average age of ~40 years old (15–17).

Recently, rTMS has gained traction as a treatment option in youth, with several meta-analysis and systematic reviews noting significant benefits and few side effects from rTMS application in youth patients (18–20). In the USA, as of April 2024, the FDA has approved rTMS as an augmentative treatment option in 15–17-year-olds, following evidence from a large systematic review and meta-analysis from eight USA studies and 1396 patients between the ages of 8 and 24 years old (21). At present, while rTMS is available for 18–25-year-olds in Australia, the effectiveness of rTMS in this population and research around safety and effectiveness is lacking. Further, RCTs are carried out in a highly controlled environment that is not generalizable to clinical practice, where high variation in patient characteristics, medication, comorbidities, and rTMS protocols are the norm.

Research evaluating treatments in a naturalistic setting holds clinical importance as it allows for greater insight into the results in real-world clinical practice. Most Australia based naturalistic studies in large clinical services and/or hospital have focused on adult populations noting 40-58% remission rates (22–25). Of these, only one pervious study, compared the effects of rTMS across age groups. While Dowling et al. (23) found that younger adults (17–25 years) demonstrated significantly higher pre-treatment depression scores compared to adults (26–64 years), and older adults (65+ years), younger adults also demonstrated significantly greater pre- to post-treatment depression score changes compared to the other groups. However, this was confounded by the fact that overall, greater depression severity was linked to better treatment response, as detailed in other TMS studies (26). Therefore, more work is needed to elucidate the effects of rTMS on different stages of brain development.

Overall, there remains under-representation of youth specific data for both efficacy and safety information when it comes to rTMS treatment. This paper aimed to further examine youth specific rTMS treatment outcomes of outpatients who received 4-7 weeks of rTMS in a private TMS service provider clinic in Perth, Western Australia. Overall, we sought to generate novel insights from Australian population data as to the efficacy, acceptability, and safety of rTMS in the youth population. Further, given the high correlation between depression, anxiety, stress, and overall quality of life (27–29), we further investigated how the treatment of depression may affect these other factors. We hypothesized that rTMS treatment would significantly improve depression (both in self-reported and clinician-rated measures), anxiety, and stress in youth. We also hypothesized that rTMS would result in significantly higher self-reported quality of life and less self-reported cognitive deficits.

## 2 Methods

### 2.1 Participants and participant inclusion

A total of 50 patients (60% female, 36% male, and 4% gender diverse) between the ages of 17 and 25 years (M = 21.22 SD = 2.22) attended out-patient rTMS treatment at one of the nine Modalis Clinics in Perth, Western Australia between January 2021 to February 2024. A total of 39 individuals received left sided 10 Hz stimulation and 11 received right sides 1 Hz stimulation. An additional breakdown of patient characteristics and outcomes per treatment protocol can be found in the **Supplementary Materials** (Sheet 3).

Patients were included in the data set for analysis if they: (1) received rTMS for an episode of major depressive disorder (MDD; as per the Diagnostic and Statistical Manual for Mental Disorders (DSM-5; APA 2013), (2) had completed both pre-treatment and post-treatment assessment measures, and (3) had current inadequate response or intolerability to current pharmacological and/or psychological treatments. Patients with psychiatric comorbidity (i.e., anxiety disorder, personality disorder, trauma) were included, if the primary condition being treated with rTMS was an episode of MDD. Patients were excluded if they had metal in the head and/or chest, were pregnant, or presented with a current brain tumor. Additional patient profiles and characteristics are described in the **Supplementary Materials** (Sheet 1 lists medication and frequency of medications participants reported to be on and Sheet 2 lists comorbidities and frequency of comorbidities in this sample).

All patients completed a minimum of 19 treatment sessions and both high-frequency and low-frequency rTMS outcomes were assessed at once for efficacy and safety due to the limited sample size. None of the 50 patients had received rTMS or ECT in the past and all had attempted and failed at least 2 previous pharmacotherapies for MDD. All participants, except for two, were taking antidepressants or at least one disorder related form of medication.

Given the naturalistic setting, medication changes were not exclusionary although treating psychiatrists avoided making significant concurrent medication changes during rTMS treatment, where possible. Other psychological and social therapies varied naturalistically during the rTMS treatment.

The University of Western Australia (UWA) Human Research Ethics Committee provided approval for access to clinical data and it’s analysis, and a Memorandum of Understanding was established between UWA, the Perron Institute, and Modalis for access to de-identified retrospective data.

### 2.2 Treatment protocol

rTMS was administered with a Therapeutics Goods Administration registered Neurosoft MS/D machine combined with The Neural Navigator (NeNa) software version 3.5 (Brain Science Tools, Utrecht, The Netherlands) for Magnetic Resonance Imaging (MRI) -guided neuronavigation. All treatment protocols and site locations were saved into the NeNa software to ensure parameter consistency across sessions. Treament was prescribed by RANZCP trained and accredited TMS psychiatrists and administered by trained and experienced TMS technicians. Neurosoft figure-of-eight coils were placed on the industry standard 45-degree angle tangential to the scalp with the handle pointing back and away from the midline (30, 31). Prior to the commencement of rTMS treatment, individual resting motor thresholds (RMT) were determined and used to prescribe rTMS treatment intensity for each individual participant by the treating psychiatrist, in accordance with standardized guidelines (RANZCP, 2018). All participants were offered either earplugs or noise cancelling headphones to minimize perception of the auditory clicks produced by the rTMS machine. A total of 35 patients received left-sides high-frequency (HF) 10 Hz dorsolateral prefrontal cortex (DLPFC) treatment (consisting of 75 trains of 40 pulses with 10 second intertrain intervals and a total of 3000 pulses), 11 received right-sided low-frequency (LF) 1 Hz DLPFC treatment (consisting of 1 train off 900 pulses for a total of 900 pulses), and 3 received bi-lateral 10 Hz left DLPFC and 1 Hz right DLPFC. Decision of treatment side was made based on clinical decision making by the prescribing psychiatrist as well as patient comfort. Both left-sided, right-sided, and bi-lateral rTMS are shown to improve symptoms of depression equally (32).

We followed standardized protocols for the identification of RMT in the motor cortex reported in past literature (31, 33). For those starting on 10 Hz left-sided DLPFC stimulation max intensity aimed for was 120% of RMT, and for the 1Hz right-sided DLPFC stimulation the maximum intensity was 110% of RMT. Overall, a total of 34 patients received left-sided stimulation at 120% RMT, 9 patients received right-sided stimulation at 110% RMT, 2 patients received left-sided stimulation at 110% RMT, 1 patient received left-sided stimulation at 105% RMT, 2 patients received right-sided stimulation at 90% RMT, 1 patient received left-sided stimulation at 90% RMT and 1 patient received right-sided stimulation at 60%.

All patients started at 80% of their RMT on the first treatment, and then increased by 5% each session (if tolerable) until the max %RMT (120%) was reached. However, if a patient found the higher intensity to be too uncomfortable and could not tolerate it, treatment intensity was capped at the intensity that they could handle (hence why some patients only reached 100% or 110% and not the maximum of 120% RMT). The titration of the %RMT was recorded in the patient’s case notes after each treatment session, to ensure consistency across sessions. In one participant intensity had to be decreased below 80% in the initial session due to discomfort. Overall, once a comfortable intensity was determined, patients did not experience any additional tolerability issues with the full course of rTMS treatment.

A total of 2 patients however switched protocol mid-treatment from right to left DLPFC. Based on treatment provider case notes, protocol switching in these 2 patients took place due to a lack of response to rTMS, or an increase in anxiety symptoms (not due to discomfort specifically in relation to rTMS) and was initiated by the treating psychiatrist’s review of the patient. Thus, as in most clinical settings where rTMS is administered with the goal to treat patients (not with research in mind), clinical judgement was used to decide on the best way to target the patients’ personal symptom presentations and comorbidities. Switching protocols in this way is supported by existing literature and both left-sided and right-sided rTMS have been found to reduce symptoms of anxiety (32, 34).

Left and right DLPFC stimulation sites were targeted using neuro navigational technology (NeNa; guided by the patient’s own structural MRI scan) for all except one patient. In patients with an MRI, a radiologist was asked to mark DLPFC locations on the acquired MRI scan to be used for neuronavigation. For the one patient without MRI, the TMS coil was placed over the left DLPFC spot which corresponds to the F3 location in the 10–20 system (35). For this participant without MRI, the coil was placed at a 45-degree angle at the identified site and the individual’s head was measured up using physical landmarks on the head and face to ensure subsequent treatment session consistency. Stimulation and other treatment parameters were recorded into the patient case notes for TMS technicians to access prior to each treatment session.

Patients received an average of 34 sessions. For those patients who were deemed as having completed a course of rTMS, the minimum number of sessions was 19 (equating to 4-weeks of treatment) and the maximum was 35 sessions (equating to 7-weeks of treatment). Sessions were carried out across a range of 32 to 92 days. This large range of days is mostly noted to be due to patients’ availability and unrelated (to their TMS course) illness.

### 2.3 Clinical assessments

Standardized and validated clinical assessments and outcome measures were used to assess changes to depression and secondary outcome measures. Assessments included the clinician-rated Montgomery-Asberg Depression Rating Scale [MADRS; (36)] collected pre and post rTMS treatment. Further, the 21-item self-report Depression, Anxiety, and Stress Scale [DASS-21; (37, 38)]. Both measures are standardized, robust assessments of depression, anxiety, and stress prevalence. For all, a higher score indicates greater frequency of symptoms. To assess for quality of life and life satisfaction, the self-reporting Quality of Life (QoL) Enjoyment and Satisfaction Questionnaires [QoLES-Q; (39, 40)] were used – where a higher score indicates greater life satisfaction and enjoyment. To assess for changes in cognition, the Cognitive Failures Questionnaire [CFQ; (41)] was used, the CFQ is a subjective, self-report measure which asks individuals to consider the everyday mistakes they make to evaluate perception, memory, and cognition – here, a higher score indicates greater frequency of cognitive ‘failures’ (i.e., forgetfulness, failures of perception).

All measures except the MADRS were assessed pre-treatment (pre-tx.), half-way through rTMS treatment (mid-tx.), and post-treatment treatment session (post-tx.). The MADRS was assessed only at pre-tx. and post-treatment timepoints. Safety and side effects of rTMS treatment were monitored across each of the treatment sessions. The MADRS and DASS-21 depression subscale were used as the primary measure for assessing depression severity and depression response. AS per past publications, responders were defined as 50% or greater reduction in MADRS and/or DASS-21 depression scores from pre-treatment to post-treatment assessment (22–26).

### 2.4 Statistical analysis

All statistical tests were set at an alpha level of p <.05. Data analysis was performed using R (version 4.0.3) with the lme4, lmerTest, and MuMIn packages for linear mixed models (LMM). Continuous variables were summarized using means and standard deviations, while categorical variables were presented as frequencies and percentages.

To examine changes in depression (both self-reported and clinician-rated), anxiety, stress, quality of life, and cognition across the treatment periods (pre-rTMS, mid-rTMS, end-rTMS), six separate LMM were used on data that was organized in a long format structure. The LMMs were specified to account for fixed effects including timepoints (coded numerically, 0 = Baseline, 2 = Middle, 4 = End) and baseline scores meanwhile, the random effect was defined as individual participants.

Model fit was assessed using marginal R² (variance explained by fixed effects) and conditional R² (variance explained by both fixed and random effects). *Post-hoc* analyses were conducted using paired-sample t-tests to assess specific pairwise differences between the three timepoints. We further calculate Cohen’s *d* to assess effect size for all statistically significant t-tests.

In addition to the primary analysis, we used IBM SPSS Statistics (version 29.0.0.0) to conduct Pearson’s r correlations to examine relationships between baseline depression and other variables including anxiety, stress, quality of life, and cognition, as well as the relationship between post-rTMS depression and these same variables. Further, we used Cohen’s r to assess effect sizes of the correlations.

## 3 Results

Of the 50 youth patients that undertook rTMS treatment, 4 individuals were excluded as there was not enough data to include them in any meaningful comparative statistical analysis, these are patients for whom data on only one timepoint was available. Noted reasons for lack of data included missed appointments or patients not completing the outcome measures. Thus, a total of 46 individuals were included in the final analysis.

Model diagnostics were performed to assess the assumptions of normality and homogeneity of variance in LMM for all six of the included variables. Further, we assessed boxplots and calculate the residuals from the model and then standardized them to assess for outliers. There were no visible outliers in the boxplots, nor any data points that had residuals greater than 3 or less than -3. The Q-Q plot of residuals indicated that the residuals were approximately normally distributed, with the data points closely following the reference line. This suggests that the assumption of normality was satisfied. This appeared to be the case for all variables with the expect of MADRS scores. However, clinical symptoms and as a result scores are generally non-linear, especially for a treatment-resistant population who are seeking rTMS intervention (inherently, a population such as this would be biased toward higher, non-normally distributed scores). Therefore, we did not control for this deviation in the associated LMM.

Additionally, the residuals *vs*. fitted values plot showed no clear patterns across any variables, implying that the assumption of homogeneity of variance (homoscedasticity) was not violated. For all variables, the residuals appeared to be randomly scattered around zero, which is a good indicator that the variance is consistent across all variables. Baseline scores across all variables were significant predictors of scores at later time points (all significant at *p* < 0.001).

### 3.1 Safety, tolerability, and reported side effects of rTMS

Approximately 52% of patients reported some side effects (see **Table 1** below), most of which cleared up within the first few sessions of treatment.

**Table 1:** Side effects of rTMS across 25 treatment sessions.

Side effect	Reported in first 1-15 sessions	Reported in last 16-35 sessions
Fatigue	24/46 (52.2%)	15/24 (62.5%)
Headache	20/46 (43.5%)	6/20 (30%)
Lightheaded	2/46 (4.3%)	0/2
Nausea	1/46 (2.2%)	0/1

There was a single case of increased suicide ideation throughout the course of treatment, however, this was not attributed to the rTMS treatment as the individual was reporting suicidal thoughts prior to the commencement of treatment. When looking only the suicide ideation items of the two scales used within the naturalistic sample reported here (DASS item number 21 and MADRS item number 10), a visible numeric decrease in the item across time can be seen (see **Supplementary Materials** Sheet 3). Overall, rTMS was well tolerated despite instances of participants having experienced mild fatigue and headaches.

### 3.2 rTMS-related changes in depression

A total of 40 self-report DASS-21 depression sub-scale were available and of these, a total of 17 (42.5%) individuals responded to rTMS based on 50% or greater reduction in scores. Meanwhile, 15 patients (37.5%) reported a decrease in symptoms ranging from 8 – 39% improvement and 8 patients (20%) did not benefit from rTMS at all. Further, a total of 27 MADRS rating scale scores were available with 11 patients (40.74%) demonstrating 50% or greater reduction in scores, 12 patients (44.44%) noting 8 – 44% improvements, and 2 patients (7.41%) demonstrating no improvement to depression scores.

**Figure 1:**
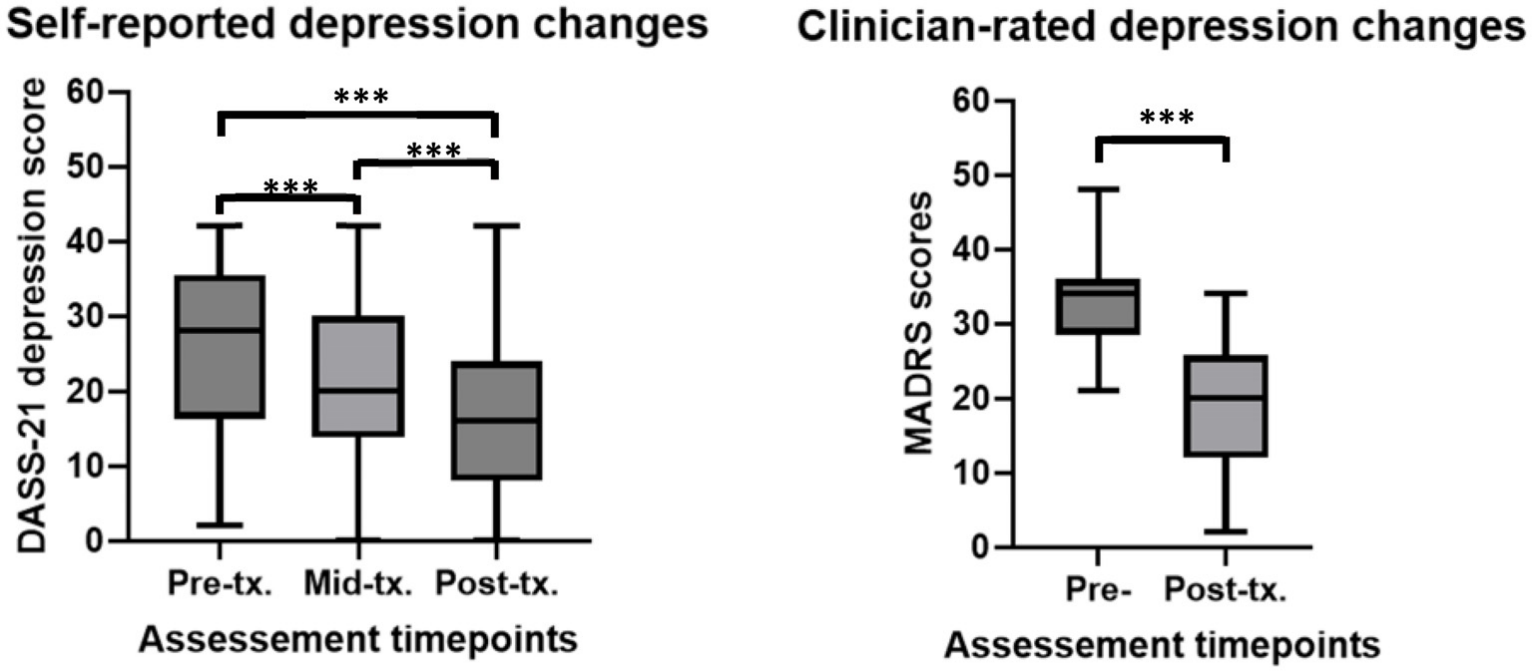
Self-report and clinician rated changes in depression before, during and after rTMS treatment in a youth population. ***significant at *p*<0.001. For DASS-21 depression scores (higher scores = more depression) at pre-tx. n = 44, at mid-tx. n = 42, at post-tx. n = 40. For MADRS (higher scores = more depression) n = 27. DASS0-21, 21-item Depression, Anxiety, Stress rating scale; MADRS, Montgomery-Asberg Depression Rating Scale; pre-tx., baseline / pre-treatment; mid-tx., mid-treatment; post-tx., final / post-treatment.

The LMM results showed that self-reported depression (DepDASS) scores decreased significantly over time (β = -2.31, t = -7.73, *p* < 0.001). Additional paired t-tests revealed significant differences between baseline and the middle time point (t(35) = 4.30, *p* < 0.001, Cohen’s *d* = 0.63), baseline and the end time point (t(35) = 6.07, *p* < 0.001, Cohen’s *d* = 0.87), and between the middle and end time points (t(35) = 3.33, *p* = 0.002, Cohen’s *d* = 0.25). See **Figure 1** for depiction of means and standard deviations. The LMM fixed effects (i.e., including timepoints and baseline scores) explained 65% of the variance in depression scores (Marginal R²), while the full model, including both fixed and random effects, explained 76% of the variance (Conditional R²). These values demonstrate that the model provided a good fit to the data.

A second LMM revealed that clinician-rated depression scores (MADRS) significantly reduced over the time course of rTMS treatment (β = -4.06, -10.59, *p* < 0.001). An additional paired t-test revealed significant differences between baseline and the end treatment time point (t(27) = 7.60, *p* < 0.001, Cohen’s *d* = 2.12). See **Figure 1** for depiction of means and standard deviations. In the LMM, both the fixed effects (Marginal R²) and the full model (Conditional R²) explained approximately 63% of the variance in depression scores, indicating a strong fit of the model to the data.

Overall, these results indicates that rTMS treatment significantly decreased and thus improved depression in youth patients.

### 3.3 rTMS-related changes in anxiety and stress

**Figure 2:**
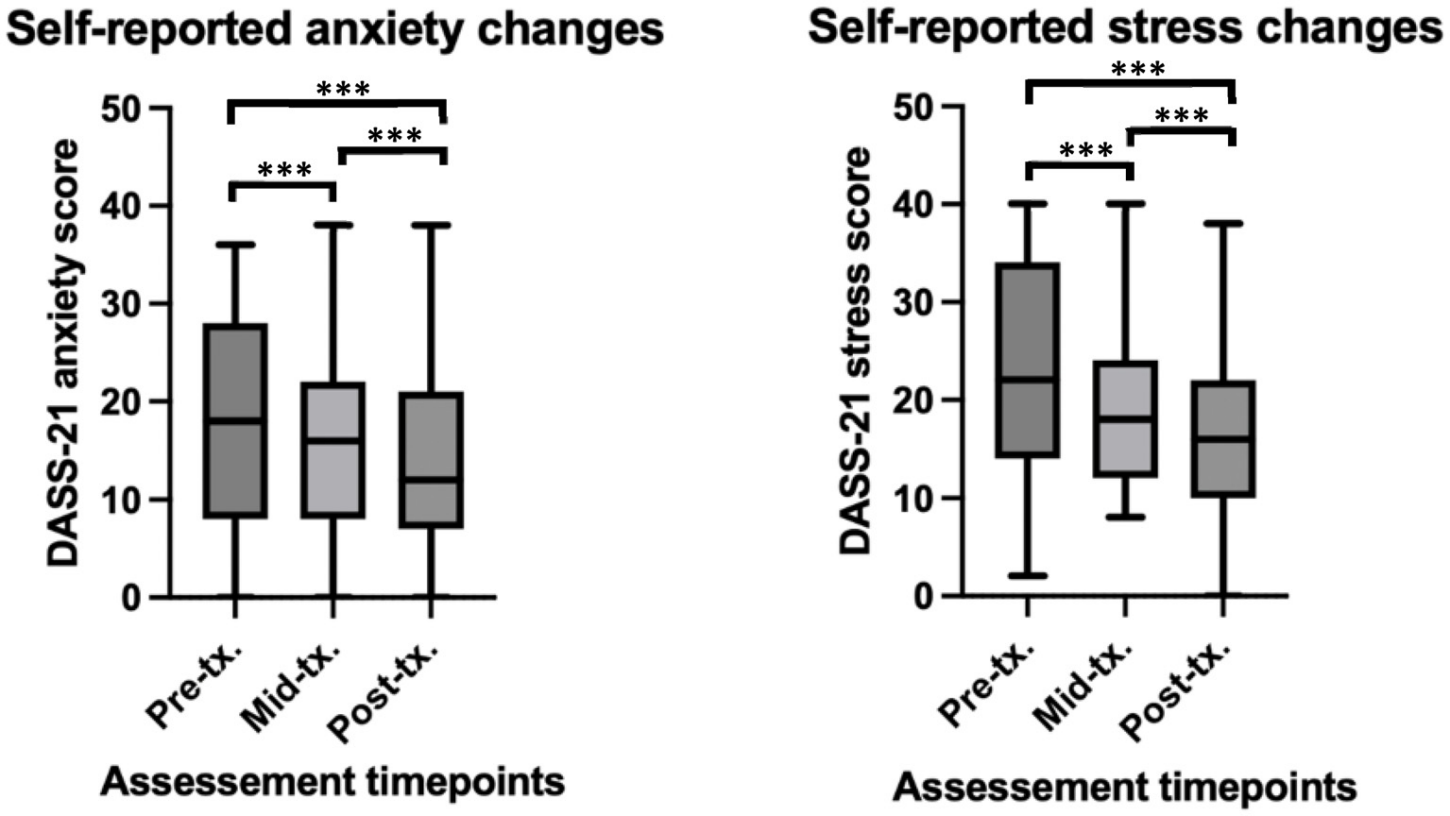
Self-report and clinician rated changes in anxiety and stress before, during and after rTMS treatment in a youth population. ***significant at *p*<0.001. For anxiety scores at pre-tx, n = 45, at mid-tx. n = 42, at post-tx. n = 41. For stress scores at pre-tx. n = 45, at mid-tx. n = 43, at post-tx. n = 41. pre-tx., baseline / pre-treatment; mid-tx., mid-treatment; post-tx., final / post-treatment.

Self-reported anxiety (AnxDASS) scores were found to significantly reduce over the course of rTMS treatment (β = -1.00, t = -4.15, *p* < 0.001). Paired t-tests revealed significant differences between baseline and the middle time point (t(36) = 2.74, *p* = 0.009, Cohen’s *d* = 0.28), and baseline and the end time point (t(36) = 3.31, *p* = 0.002, Cohen’s *d* = 0.39). No significant difference was found between the middle and end time points (t(36) = 1.45, *p* > 0.05). See **Figure 2** for depiction of means and standard deviations. The LMM fixed effects explained approximately 75% of the variance in anxiety scores (Marginal R²), while the full model accounted for 80% of the variance (Conditional R²), indicating a strong fit of the model to the data.

Self-reported stress (StrDASS) scores were found to significantly reduction over the rTMS treatment time course (β = -1.68, -6.38, *p* < 0.001). Paired t-tests revealed significant differences between baseline and the middle time point (t(36) = 3.66, *p* < 0.001, Cohen’s *d* = 0.47), baseline and the end time point (t(36) = 5.48, *p* < 0.001, Cohen’s *d* = 0.72), and a significant difference between the middle and end time points (t(36) = 2.26, *p* = 0.03, Cohen’s *d* = 0.29). See **Figure 2** for depiction of means and standard deviations. The LMM fixed effects explained approximately 71% of the variance in stress scores (Marginal R²), and the full model accounted for 72% of the variance (Conditional R²), indicating a strong fit of the model to the data.

Overall, this indicates that rTMS treatment significantly decreased and thus improved anxiety and stress in youth patients.

### 3.4 rTMS-related changes in quality of life and cognition

**Figure 3:**
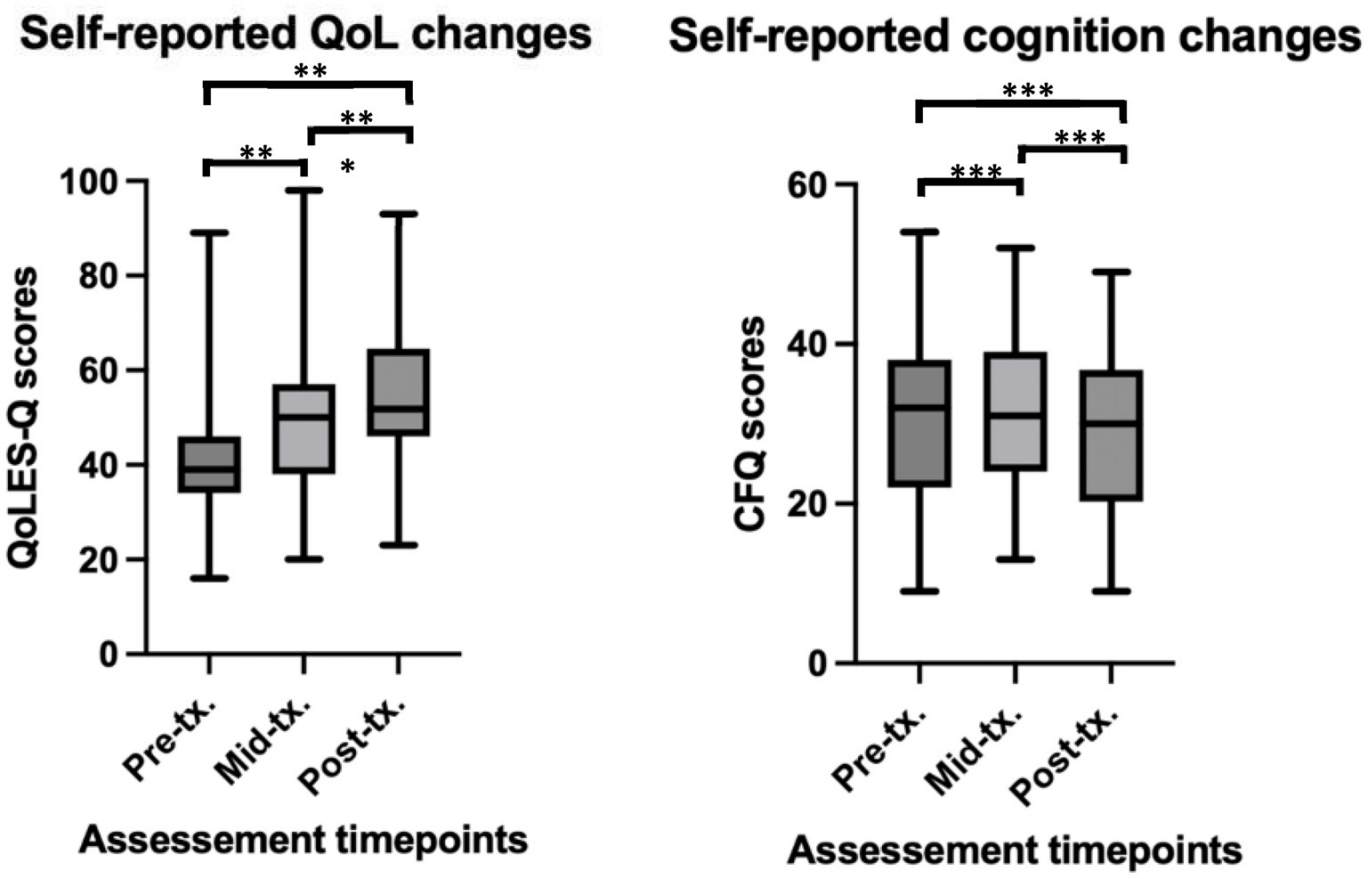
Self-reported changes in QoL and cognition before, during and after rTMS treatment in a youth population. **significant at p<0.01, ***significant at p<0.001. For QoL scores (higher scores = better QoL) at pre-tx. n = 45, at mid-tx. n = 43, at post-tx. n = 41. For CFQ scores (higher scores = worse cognition), at pre-tx. n = 44, at mid-tx. n = 43, at post-tx. n = 39 – higher scores on the CFQ indicate worse cognitive performance. QoL, quality of life; QoLES-Q, Quality of Life Enjoyment and Satisfaction Questionnaire; CFQ, cognitive failures questionnaire; pre-tx., baseline / pre-treatment; mid-tx., mid-treatment; post-tx., final / post-treatment.

Baseline self-report QoL scores were found to significantly increases over time (β = 3.49, t = 5.94, *p* < 0.001), suggesting that individuals’ self-reported QoL scores improved as treatment progressed. Paired t-tests revealed significant differences between baseline and the middle time point (t(36) = -3.28, *p* = 0.002, Cohen’s *d* = -0.62), and baseline and the end time point (t(36) = -4.73, *p* < 0.001, Cohen’s *d* = -0.86). No significant difference was found between the middle and end time points (t(36) = -1.68, *p* > 0.05). See **Figure 3** for depiction of means and standard deviations. The LMM fixed effects explained approximately 42% of the variance in QoL scores, and when both fixed and random were considered, the model accounted for 53% of the variance in QoL scores. Overall, this suggesting a moderately strong fit to the data.

Finally, significant decreases in self-reported cognitive ‘failure’ symptoms over time (β = -0.64, t = -2.43, *p* = 0.01) was also found. Paired t-tests revealed no significant differences between baseline and the middle time point (t(35) = -0.27, *p* > 0.05), and baseline and the end time point (t(35) = -1.94, *p* = 0.05), however, a significant difference was found between the middle and end time points (t(35) = 2.44, *p* = 0.02, Cohen’s *d* = -0.22). See **Figure 3** for depiction of means and standard deviations. The LMM fixed effects explained a substantial portion of the variance in CFQ scores, with fixed effects explaining 76% of the variance in scores, and the full model explaining 78% of the variance.

Overall, these results indicate that rTMS treatment significantly decreased reporting of self-reported cognitive ‘failures’ and improved QoL.

### 3.5 Relationships between depression, and anxiety, stress, QoL, and cognition

A Person’s r test between baseline DASS-21 depression and the MARDS did not find a statistically significant correlation between the two measures of depression. Indicating possibly that patients and clinicians rate depression differently. MADRS was not associated with any other measure at baseline including measures of anxiety, stress, QoL or self-reported cognition.

**Figure 4:**
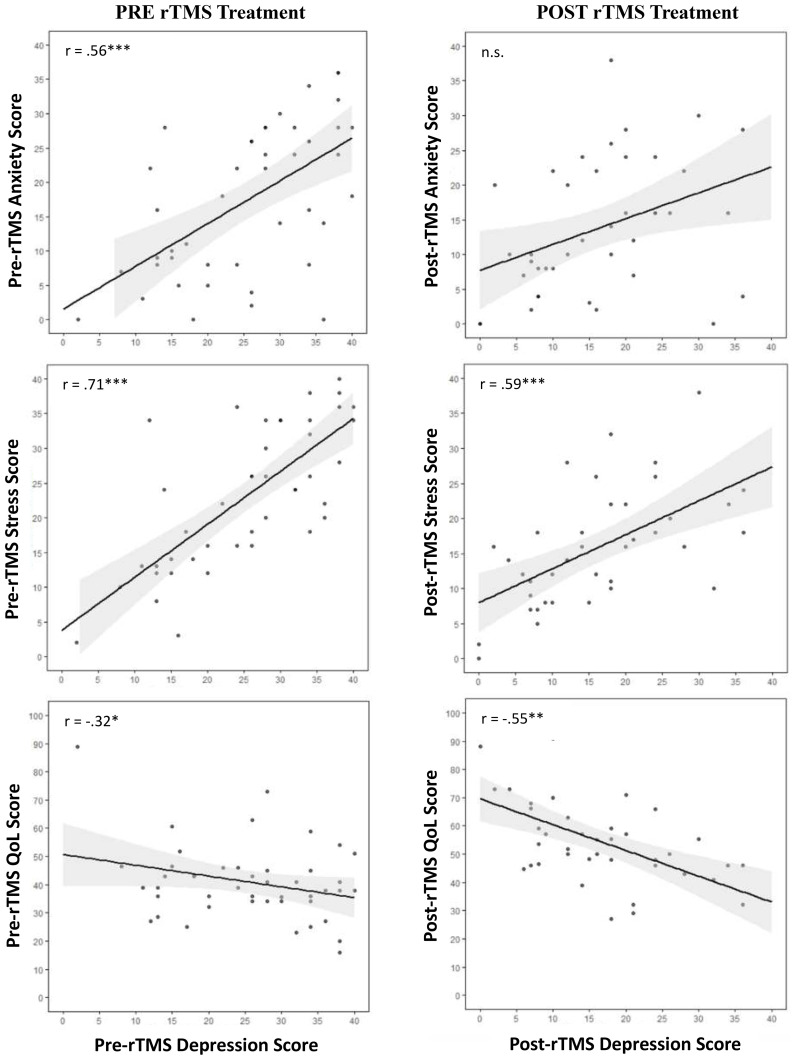
Correlation scatterplots with line of best fit and 95% confidence intervals for the relationships between pre and post treatment depression scores with anxiety, stress, QoL, and subjective cognition. *p < 0.05; ** *p* < 0.01; *** *p* < 0.001. Anx, anxiety; Dep, depression, QoL, Quality of Life, pre-tx., pre-treatment; post-tx., post-treatment.

A significant positive relationship was observed between baseline DASS-21 depression scores and both anxiety (r = .56, *p* < 0.001, Cohen’s *d* = 0.81) and stress (r = .71, *p* < 0.001, Cohen’s *d* = 0.32). Further, baseline DASS-21 depression was significantly and negative correlated to baseline QoL (r = -.32, *p* = 0.03, Cohen’s *d* = -1.17) but not with cognitive failure symptoms (**Figure 4**).

On the other hand, end-treatment DASS-21 depression was significantly positively correlated with stress (r = .59, *p* < 0.001, Cohen’s *d* = 0.10) but not anxiety. Further, post-treatment DASS-21 depression was significantly, negatively correlated with post-treatment QoL (r = -.63, *p* < 0.001, Cohen’s *d* = -2.74) but not with cognitive failure symptoms (**Figure 4**).

Interestingly, end-treatment MADRS was significantly negatively correlated with self-reported end-treatment anxiety (r = -.45, *p* = 0.02, Cohen’s *d* = 0.57), as well as with stress (r = .50, *p* = 0.009, Cohen’s *d* = -0.33), QoL (r = -.55, *p* = 0.004, Cohen’s *d* = -2.71) and cognitive failure symptoms (r = .40, *p* = 0.04, Cohen’s *d* = 1.05) (**Figure 4**).

## 4 Discussion

To the best of our knowledge, this is the first naturalistic paper in Australia to assess the acute rTMS treatment outcomes from an out-patient private TMS service provider clinic, in youth presenting with a current MDD episode. As hypothesized, our paper found a significant improvement in self-reported and clinician-rated depression in youth who received rTMS treatment. Overall, 80% of patients self-reported a benefit from rTMS treatment. Specifically, the response rate (defined by a 50% reduction in depression scores) of 42.5% appears to be mostly consistent with the range of response reported in other studies assessing rTMS efficacy in both adults [~46–68%; (26, 42)] and youth [~41–56%; (43, 44)]. However, our observed response rates may be considered slightly lower than in previous published works on youth, likely due to the naturalistic nature and the less controlled, more varied treatment parameters and clinical characteristics of the included patients.

The paper revealed that rTMS treatment was generally well tolerated with some transient mild headaches and fatigue, as well as rare occurrences of light-headedness. This is encouraging, and consistent with side-effects reported in other TMS research in adults (45, 46) and in youth (18–20). There were no documented seizures or increases in suicide ideation due to rTMS treatment. Most side effects appeared to significantly improve or resolve after the first two weeks of treatment and were not influential enough to warrant cessation of treatment by the patient or treating psychiatrist.

Interestingly, when considering only the suicide ideation items of the two scales used within the clinic (DASS and MADRS), a visible numeric decrease in the item across time can be seen. Indeed, research has demonstrated TMS’ efficacy in reducing suicide ideation in both adult (47) and adolescent/youth populations (48). However, how these decreases in suicide ideation interact with rTMS antidepressant effect is yet to be more comprehensively investigated.

For the most part we see significant changes in symptoms between the baseline and mid-treatment (first 10-15 sessions) timepoints, and fewer significant differences between mid-treatment and end-treatment timepoints. However, symptoms continue to decrease from mid- to end- treatment. This could therefore suggest that the most changes in symptoms occur in the first 10-15 sessions of treatment, which seems to be in line with come recently published TMS work with adolescent populations (49). This was true for both depression, anxiety, and stress symptoms.

It has been estimated that approximately 65-85% of patients with depression also experience symptoms of anxiety (27, 50). In addition to significant reductions in depression, this paper revealed that comorbid anxiety and stress also improved post-treatment. This is consistent with the literature in pharmacological (51) and rTMS studies (28, 52, 53), and in naturalistic (22–25) and clinical trial (16, 54) studies, where improvements in depression are often accompanied by improvements in anxiety. Within this youth sample, a reduction in anxiety was observed in 69% of participants, somewhat higher than what is reported in the literature [~48%-61% of participants improved; (16, 28)].

Further, we found that pre-treatment depression significantly related to pre-treatment anxiety, but this relationship was no longer significant at the post-treatment timepoint. If anxiety and depression are highly correlated, as the literature suggests, we would expect their relationship to be maintained even after treatment. While this was the case for MADRS and the DASS anxiety sub-scale, there was no significant relationship between self-reported depression and anxiety on the DASS post-treatment. One reason for a lack of significant relationship at this post-treatment timepoint may be that rTMS is having more effect on one disorder than the other. However, the therapeutic mechanisms of rTMS in depression and anxiety remain poorly understood (55, 56). Alternatively, the different results in clinician-rated and self-report post-treatment measures could be due to variation in perception of symptoms between patient and clinician (57). The increase in the variability and spread of reported symptoms at the post-treatment timepoint for both depression and anxiety may be due to the small sample size or may reflect individual differences in symptoms and symptom presentation. Nonetheless, there is a clear effect of rTMS on both anxiety and depression in youth within this sample. However, given the limited sample size, this finding must be further validated and explored.

Interestingly, self-reported stress improved as a result of rTMS within this sample. The specific dimension of stress assessed within the used DASS scale is defined as perceived stress, which is characterized primarily by symptoms of tension, inability to relax, irritability, fidgety or jumpy, and/or easily upset, symptoms which overlap and are often associated with anxiety, as well as situational/external demands. Thus, while rTMS may be affecting general life stress the participant may have experience outside of seeking treatment, it is also possible that the drop in stress could be attributed to reduced stress around seeking treatment/receiving the rTMS intervention rather than the rTMS intervention itself. Therefore, it may be difficult to understand the nature of the relationship between stress and depression. Nonetheless, a significant, positive relationship between stress and depression that was maintained at both the pre- and post- treatment time points, as has been reported for antidepressant medication treatment (29). Depression, anxiety and stress are highly correlated (58) suggesting a need for future research to investigate these disorders independently, and their responsiveness to rTMS treatment.

Our findings further indicate that the reduction of depressive symptoms following rTMS is accompanied by an improvement in QoL. Specifically, our results show a significant increase in QoL as well as a persistent negative correlation between both pre- and post-rTMS depression and pre- and post-rTMS QoL, respectively. Thus, lower levels of depression related significantly to higher levels of QoL. This finding is supported by broad evidence for a relationship between improvements in depression (no matter the treatment type) and QoL (59–62). This is an important observation, indicating that effective treatment can induce an improvement in not only depression but also overall well-being in a relatively short timeframe (i.e., 4-7 weeks). As far as we are aware, this is the first paper to look at QoL following rTMS treatment specifically. While there was a significant improvement in QoL before and after rTMS which related to reductions in depressive symptoms, the effect appeared to be moderate and thus requires further investigation. Better understanding of the relationship between QoL, rTMS, and symptom improvement could provide valuable insights into the broader impact of rTMS treatment in youth.

Approximately 40% of patients with depression have been found to experience cognitive impairments (63). In our sample, rTMS improved subjective cognition, which aligns with the results of meta-analyses noting modest effect size improvements in cognition in active as compared to sham rTMS treatment (64, 65). While some studies assessing different treatment modalities report a correlation between improvements in self-reported cognition and depression symptoms (66), this is not a consistent effect (63), and not one we see in our youth sample. However, many of the published results pertaining to cognition, depression, and the effects of different treatments, are based on standardized tests of cognition and cognitive control, whereas we assessed subjective, self-report measures. In addition, it may be that a larger sample size is needed to detect robust, significant differences in cognition. Future research should aim to further explore the relationship between self-report measures of cognition and symptoms of depression.

Limiting our analysis, certain details around patient history (e.g., duration of episode, diagnosis date etc.) were not available as this data was extracted from an outpatient rTMS service provider and de-identified records. In addition, due to missing data, some of the comparisons (i.e., MADRS pre- to post- treatment) are underpowered and much of the presented results relied on self-reported measures. Not all patients completed the full recommended 35 sessions, and not all completed treatment within the recommended 4-7 weeks. For some, treatment took as long as 13 weeks, due to patient-related life circumstances. While patients were allocated across three rTMS protocols (HF-left DLPFC, LF-right DLPFC, or sequential bilateral rTMS), due to the naturalistic design and limited sample size, we were unable to perform comparisons between the different protocols. Further, in this paper, we were not able to obtain follow-up data as to the longer-term effects of rTMS treatment on depression in youth. This is a limitation of rTMS clinical research overall and an important topic that needs to be addressed in future studies.

Finally, there was variability in including stimulation intensity ranging from 60% to 120% RMT, as well as wide range in treatment duration (32 to 92 days), session numbers (19 to 35), and treatment sides which can interpretation of the results challenging but is normal in naturalistic settings and is important to report. Importantly left-sided, right-sided, and bilateral rTMS are all found to produce equal effects on depression and anxiety in the adult rTMS literature (32, 34). The recommendations for intensity of TMS stimulation are highly varied with few studies comparing the outcome of antidepressant effect across intensities. Basic research tells us that intensities anywhere from 50-140% can produce neuromodulatory effects (67–69). Meanwhile, most studies demonstrate that on average ~20 treatment sessions are enough to produce a moderate decrease in depressive symptoms, with a recently published large scale analysis of TMS outcomes in youth demonstrating a trajectory of improvement over the first 10 sessions, during which the greatest symptom reductions were observed (49). Evidence as to the effects of different protocols in youth are limited and more research is needed to explore this.

Notwithstanding these limitations, this paper was able to demonstrate significant clinical outcomes in data collected in a naturalistic setting, which reflects real world practice, where high rates of psychiatric comorbidity, varied current medication use, and treatment resistance is the norm. Therefore, providing a promising, initial account of rTMS use in youth populations and calling for further, more rigorous investigation.

Future research should aim to assess and report on larger youth patient populations with longer term follow-ups, as well as compare efficacy of different rTMS treatment protocols. Additionally, it will be important to compare youth response rates to adult response rates in naturalistic clinical settings. Further, future research should more robustly assess for potential confounders (i.e., the impact of concurrent medications and psychological treatments, previous treatment history, comorbid conditions, etc.) and how they might influence the outcomes of rTMS treatment in youth. While this paper is not able to do so due to sample size and the naturalistic nature, despite the high variability in variables, patient characteristics, and treatment protocols we note that rTMS is still effective. Finally, our results highlight the need to standardize and incorporate measures of QoL and cognition into rTMS research and clinical practice to better assess patient outcomes.

To conclude, the findings of this naturalistic report suggest that an acute course of rTMS provided in a private clinical setting results in similar response rates to the existing rTMS literature in the youth population. The paper notes significant improvements in anxiety, stress, QoL and cognition, indicating that rTMS treatment can produce an improvement in overall well-being in a relatively short timeframe. While limited by a small sample size and self-report measures, this paper adds to the growing body of research showing that rTMS is an important therapeutic option in real-world practice for treating MDD in Australian adolescent and youth populations. Further clinical implementation and research into the efficacy of rTMS in youth populations is warranted.

## Data Availability

The de-identified datasets analyzed for this study can be obtained upon request from the corresponding author.
